# Role of NADPH Oxidase-Induced Hypoxia-Induced Factor-1*α* Increase in Blood-Brain Barrier Disruption after 2-Hour Focal Ischemic Stroke in Rat

**DOI:** 10.1155/2021/9928232

**Published:** 2021-08-14

**Authors:** Yanping Wang, Yufei Shen, Xin Yu, Jingxia Gu, Xiaoling Zhang, Beiqun Zhou, Yanyun Sun, Congying Xu, Shuxia Qian

**Affiliations:** ^1^Department of Neurology, The Second Hospital of Jiaxing City, Jiaxing, 314000 Zhejiang, China; ^2^Bengbu Medical College (Department of Neurology, The Second Hospital of Jiaxing City), China; ^3^Institute of Neuroscience, Soochow University, Suzhou 215123, China

## Abstract

We recently showed that inhibition of hypoxia-induced factor-1*α* (HIF-1*α*) decreased acute ischemic stroke-induced blood-brain barrier (BBB) damage. However, factors that induce the upregulation of HIF-1*α* expression remain unclear. Nicotinamide adenine dinucleotide phosphate (NADPH) oxidase played a critical role in reperfusion-induced BBB damage after stroke. However, the role of NADPH oxidase in BBB injury during the acute ischemia stage remains unclear. This study is aimed at investigating the role of NADPH oxidase in BBB injury and the expression of HIF-1*α* after acute ischemic stroke. A sutured middle cerebral artery occlusion (MCAO) model was used to mimic ischemic stroke in rats. Our results show that the inhibition of NADPH oxidase by apocynin can significantly reduce the BBB damage caused by 2 h ischemic stroke accompanied by reducing the degradation of tight junction protein occludin. In addition, treatment with apocynin significantly decreased the upregulation of HIF-1*α* induced by 2 h MCAO. More importantly, apocynin could also inhibit the MMP-2 upregulation. Of note, HIF-1*α* was not colocalized with a bigger blood vessel. Taken together, our results showed that inhibition of NADPH oxidase-mediated HIF-1*α* upregulation reduced BBB damage accompanied by downregulating MMP-2 expression and occludin degradation after 2 h ischemia stroke. These results explored the mechanism of BBB damage after acute ischemic stroke and may help reduce the associated cerebral hemorrhage transformation after thrombolysis and endovascular treatment after ischemic stroke.

## 1. Introduction

The blood-brain barrier (BBB) which is composed of microvascular endothelial cells, astrocytes, neurons, pericytes, and basement membrane could prevent blood components from entering the brain parenchyma and maintain the basic stability of the brain environment. Chronic stress could induce anxiety, depression, and schizophrenia, and stress could also damage the BBB integrity [1]. Previous studies have shown that BBB plays important roles not only in mental illnesses such as schizophrenia, autism, and depression [2] but also in neurological diseases such as stroke and dementia [3].

After acute ischemic stroke, protecting the BBB is a promising strategy [4, 5] to decrease cerebral hemorrhage, the most feared complication in patients with intravenous tissue plasminogen activator (tPA) thrombolysis [6] or postendovascular treatment [7]. BBB damage within the reperfusion stage has been investigated widely as the restoration of ischemic cerebral blood flow is essential for the occurrence of the most devastating results of BBB damage: hemorrhage transformation and edema [8–10]. However, the mechanism of BBB disruption during the acute phase of ischemia, especially within the time window of thrombolysis [11], requires a lot of research.

Hypoxia-inducible factor-1 alpha (HIF-1*α*) expression upregulation was observed in noninfarcted ventromedial striatum and preoptical area (POA) where BBB damage was observed after 2 h MCAO [12], and our recent study reported that YC-1, a HIF-1*α* inhibitor, decreased BBB damage by regulating matrix metalloproteinase-2 (MMP-2) and vascular endothelial growth factor (VEGF) during acute cerebral ischemia [13]. However, the factors that induce the upregulation of HIF-1*α* in the acute phase of ischemic stroke remain unclear.

Acidosis and free radicals have been shown to upregulate HIF-1*α* expression [14], and oxidative stress plays a critical role in BBB disruption during the reperfusion phase after ischemic stroke [15]. For example, nicotinamide adenine dinucleotide phosphate (NADPH) oxidase was shown to play a key role in reperfusion-induced BBB damage in experimental stroke [16], and inhibition of NADPH oxidase is neuroprotective after ischemic stroke [17]. In addition, apocynin, a NADPH oxidase inhibitor, significantly improved the endothelial function of rat and human blood vessels [18]. However, the role of NADPH oxidase in BBB injury within the thrombolytic time window of acute ischemic stroke remains unclear.

In the current study, a rat middle cerebral artery occlusion (MCAO) model was used to mimic ischemic stroke. The effect of NADPH oxidase inhibition on BBB injury and HIF-1*α* expression after acute ischemic stroke will be explored.

## 2. Material and Methods

### 2.1. Animal Model of Middle Cerebral Artery Occlusion

**Figure 1:**
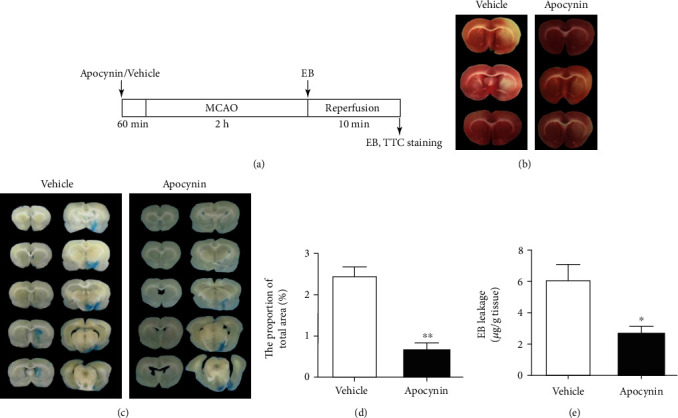
Effect of apocynin treatment on BBB injury induced by 2 h ischemia. (a) The experimental procedure diagram. (b) TTC staining showed the success of MCAO surgery. (c) Ten consecutive brain coronal sections demonstrated leakage of Evan's blue (EB) in the rats treated with vehicle or apocynin. (d) EB extravasation is quantitated and expressed as the average area ratio (%) of the measured brain slice section (%). ^#^*P* < 0.05 versus the vehicle ctrl, *n* = 6 per group. (d) Another method to quantitatively determine the amount of EB leakage in brain tissue, and it was expressed as per gram of brain tissue (*μ*g/g) by the external EB standard curve. ^#^*P* < 0.05 versus vehicle ctrl. Data were demonstrated as the mean ± SEM, *n* = 5 per group.

Thirty-four male Sprague-Dawley rats were purchased from SLAC Company (Shanghai, China). Under the condition of constant temperature (23 ± 1°C) and light-controlled vivarium (12-hour light/12-hour dark cycle), 2-3 rats are housed in each cage. Rats can get water and food for free. The Soochow University Animal Care University Committee approved animal procedures in accordance with the National Institutes of Health Laboratory Animal Care and Use Guidelines. Each effort is to reduce the number of animals and minimize their suffering. Rats (270-290 g body weight) underwent a 2-hour MCAO operation using a suture model. We followed the research method of Shen et al. in 2018 [13]. The success of the operation was further tested by 2,3,5-triphenyltetrazolium chloride (TTC) staining (Figure 1).

### 2.2. Apocynin Administration

Apocynin (Sigma) or vehicle was recruited to inhibit NADPH oxidase. It was dissolved in 1% DMSO, and 40 mg/kg body weight was given via femoral vein one hour before MCAO surgery. This dose has been shown to effectively inhibit NADPH oxidase and reduce BBB damage after stroke [16].

### 2.3. Evan's Blue Leakage Detection

Evan's blue (EB) dye extravasation is a reliable and widely used method to detect BBB injury. After 2 hours of MCAO, EB was injected through a tail vein (Sigma, St. Louis, MO, USA, 2% wt/vol in PBS, 3 mL/kg). After 10 minutes of reperfusion, the EB was fully circulated to the ischemic hemisphere; then, ice-cold PBS was perfused, and the brain was quickly removed [19]. We detect the severity of BBB damage through either quantifying the mean leakage area which was calculated as averaged area proportion of the sections measured [20] or measuring content in the tissue of nonischemic and ischemic brain hemisphere [21].

### 2.4. Immunofluorescence Staining

The rats were immediately perfused with PBS and 4% PFA after cerebral ischemia for 2 hours. We followed the previously published methods of Shen et al. in doing immunofluorescence staining [13]. In brief, 20 *μ*m thick cryosections were preincubated for 1 hour at room temperature in PBS which contained 0.1% Triton X-100, 1% BSA, and 5% goat serum (Solarbio, Beijing, China) to cover nonspecific binding sites. The HIF-1*α* (1 : 200, Abcam) and RECA-1 (1 : 100, Abcam) primary antibodies were applied to the brain slices and incubated overnight at 4°C. Appropriate secondary antibodies that bind to Cy3 (anti-mouse, 1 : 800) or 488 (anti-rabbit, 1 : 800) were used for detection. The nucleus was stained with Dapi. The LSM 700 confocal laser scanning microscope (Zeiss) was used to take images from the ischemic area and the mirror nonischemic area [13].

### 2.5. Western Blot

We did this experiment following a recently published paper [21]. Briefly, after collecting the tissue of the ventral striatum and preoptic area (region of interest 1, ROI 1) as well as the cortex and dorsal striatum (region of interest 2, ROI 2) of the ischemic (I) and nonischemic (NI) hemispheres, we detected the protein concentration using a BCA protein detection kit (Beyotime, Haimen, Jiangsu, China). After boiling the aliquots of the homogenate (30 *μ*g total protein), they were electrophoresed on a 10% SDS-PAGE acrylamide gel and then transferred to a 0.45 *μ*m PVDF membrane (Millipore, Billerica, Massachusetts, USA). After blocking the membrane in PBS-T (phosphate-buffered saline and 0.1% Tween-20) containing 5% skim milk for 2 hours, it was incubated overnight with occludin (1 : 300, Invitrogen), HIF-*α* (1 : 300, Novus), or *β*-actin (1 : 5000, Hubei, China) primary antibodies. After washing three times with PBS-T, incubation with horseradish peroxidase- (HRP-) conjugated anti-rabbit or anti-mouse secondary antibody (Boster, Wuhan, Hubei, China) followed for 2 hours at room temperature. The membrane was developed and photographed using the Super Signal West Pico HRP substrate kit (Thermo Fisher, Rockford, IL, USA). The protein band intensity was quantified after normalization with *β*-actin. Every measurement is repeated three times.

### 2.6. Zymography

The tissues of the ischemic (I) and nonischemic (NI) brain hemispheres are homogenized in the lysis buffer for matrix metalloproteinase, and the level of MMP-2/9 in the homogenate was determined by the gel-gelatin zymography method [13].

### 2.7. Statistical Analysis

The data were shown as the mean ± SEM. Statistical analysis adopts one- or two-way analysis of variance (SPSS software, version 17.0). The value of *P* < 0.05 is statistically significant.

## 3. Results

### 3.1. Apocynin Treatment Alleviates BBB Injury and Degradation of Tight Junction Protein Occludin Induced by 2-Hour MCAO

We first explored whether inhibiting NADPH oxidase could decrease the disruption of BBB integrity induced by 2 hours of MCAO. The experimental program diagram is shown in Figure 1(a). Apocynin was administered 60 min before 2 h MCAO. EB was recruited to determine BBB permeability [22]. We first used TTC staining to confirm the success of our MCAO surgery (Figure 1(b)). A set of representative images of EB dye in the sliced brain is provided in Figure 1(c). Extravasation of EB was obviously seen in the ROI 1 of ipsilateral cerebral hemisphere of rats subjected to 2-hour MCAO (Figure 1(c)). Treatment with NADPH oxidase inhibitor apocynin dramatically reduced the EB leakage area (Figure 1(c)), and quantitative data demonstrated that apocynin reduced EB extravasation by approximately 75% (Figure 1(d)), indicating that inhibition of NADPH oxidase can effectively reduce BBB damage within 2 hours after the onset of ischemia stroke.

**Figure 2:**
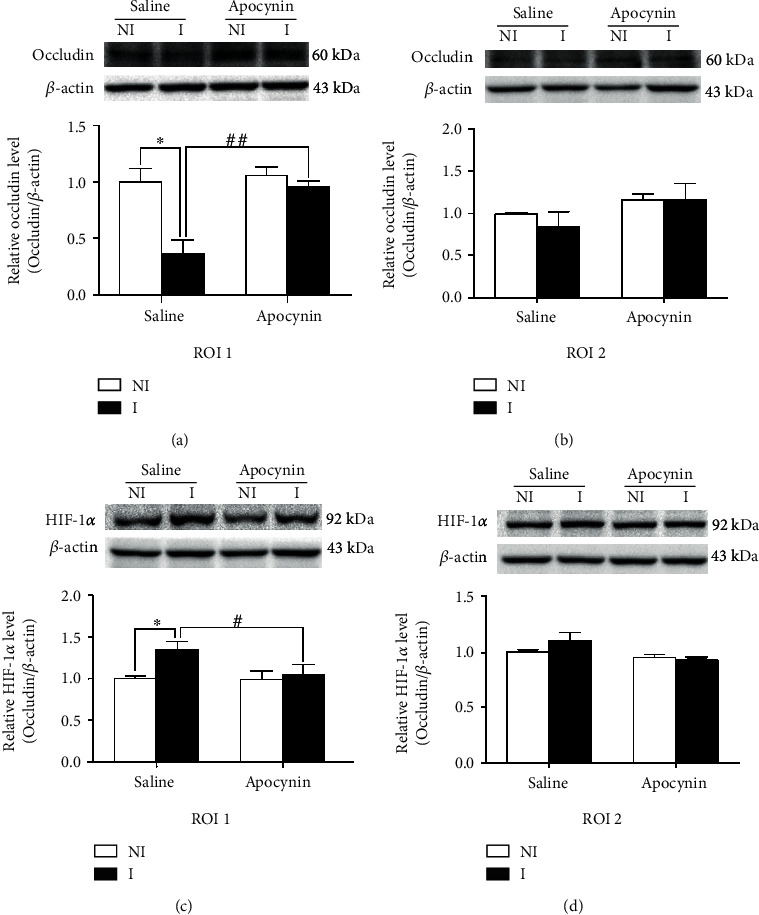
Effect of apocynin on occludin degradation and HIF-1*α* upregulation after 2 h MCAO. Western blot was recruited to detect occludin expression in nonischemic (NI) and ischemic (I) brain tissues. (a, b) Representative blots showed expression of occludin in regions of interest (ROI) 1 (a) and 2 (b) in apocynin- or vehicle-treated rats (upper panel). After the normalization, the relative band intensity of occludin to *β*-actin was quantified (lower panel). After two hours of MCAO, the occludin level in ROI 1 was significantly reduced (^∗^*P* < 0.05, compared with the vehicle group in ROI 1), while the occludin level in ROI 2 did not significantly decrease (*P* > 0.05). Apocynin treatment significantly alleviated the degradation of occludin (*P* < 0.05). (c, d) Representative blots demonstrated the expression of HIF-1*α* in ROI 1 (c) and ROI 2 (d) of apocynin- or vehicle-treated rats (upper panel). HIF-1*α* expression was quantified after normalization to *β*-actin (bottom panel). The expression of HIF-1*α* in ROI 1 (^∗^*P* < 0.05) but not in ROI 2 (*P* > 0.05) was significantly increased in the vehicle group after two hours of MCAO. Apocynin treatment significantly reduced the HIF-1*α* increase (*P* < 0.05). The data are expressed as the mean ± SEM, *n* = 5 per group.

Degradation of occludin is a key factor in BBB injury after 2 hours of MCAO [21]. In order to check if the upregulation of free radicals degraded the occludin, we used western blot to examine the expression of occludin in the interest region. We demonstrated that after 2 hours of ischemia, occludin degradation was seen in the ventral striatum and preoptical area (ROI 1, Figure 2(a)), but not in the cortex and dorsal striatum (ROI 2, Figure 2(b)). Pretreatment with apocynin significantly decreased occludin degradation induced by 2 hours of MCAO in ROI 1 (Figure 2(a)), indicating that NADPH oxidase had a key role in the degradation of occludin after 2 hours of ischemia stroke.

### 3.2. Effect of Apocynin on 2 h MCAO-Induced HIF-1*α* Expression

Upregulated HIF-1*α* has been shown to contribute to the disruption of BBB integrity after 2 h MCAO [12, 13]. In this study, we tested whether inhibition of NADPH oxidase could reduce BBB damage through downregulating HIF-1*α*. Our results showed that after 2 hours of ischemia, HIF-1*α* in ROI 1 was significantly upregulated (Figure 2(c)), while HIF-1*α* in ROI 2 was not upregulated (Figure 2(d)), and inhibition of NADPH oxidase with apocynin could significantly prevent this increase (Figure 2(c)), indicating that NADPH oxidase played an important role in HIF-1*α* upregulation induced by 2 hours of ischemia stroke.

### 3.3. Effect of Apocynin on MMP-2 Expression after 2 h Ischemia

**Figure 3:**
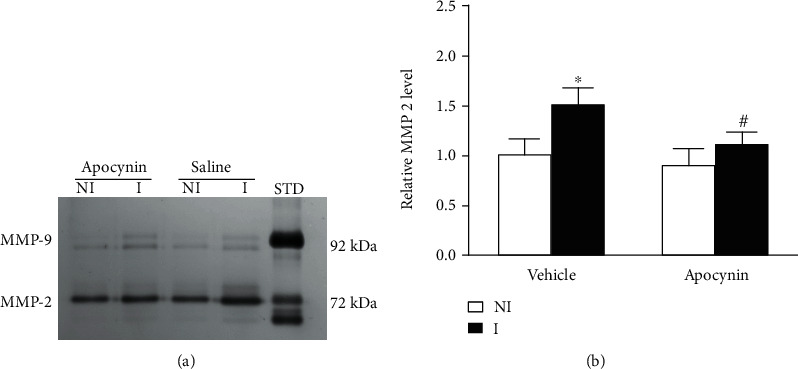
Effect of apocynin on 2 h ischemia-induced MMP-2 upregulation. After 2 hours of ischemia, the expression of MMP-2/9 was induced in the ischemic brain tissue. (a) Representative gelatin zymogram demonstrated the MMP-2/9 levels in the brain tissue of nonischemic (NI) and ischemic (I) sites. The MMP-2 level was quantitated (b). After 2 hours of ischemia, MMP-2 in ischemic tissues increased significantly (compared to NI, *P* < 0.05, *n* = 5), and apocynin treatment can significantly prevent the increase of MMP-2 (compared to vehicle, *P* < 0.05, *n* = 5). Data were expressed as the mean ± SEM.

The MMP-2/9 level after 2 hours of MCAO was detected by gelation zymography (Figure 3). In the nonischemic (NI) hemisphere, the levels of MMP-2 were not significantly different, and the results of MMP-9 were similar (Figure 3(a)). Consistent with our previous study, in the ischemic hemisphere (I), the level of MMP-2 increased significantly after 2 hours of MCAO (Figure 3), and treatment with apocynin significantly prevented the upregulation of MMP-2 induced by 2 hours of MCAO (Figure 3(b)), suggesting that NADPH oxidase was critically involved in HIF-1*α*-mediated MMP-2 upregulation by 2 hours of ischemia stroke.

### 3.4. Expression of HIF-1*α* in Blood Vessel after 2 Hours of MCAO

**Figure 4:**
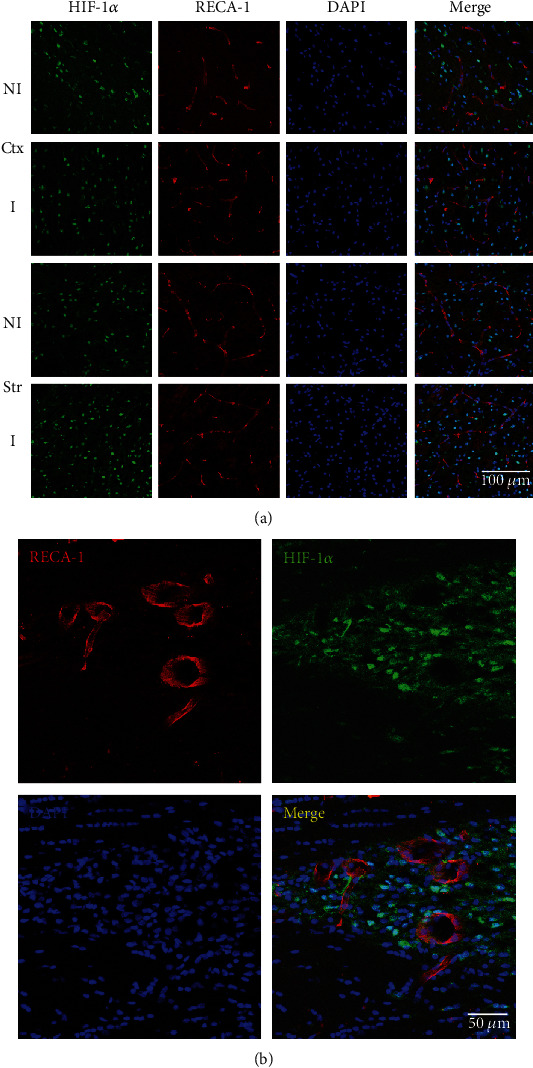
Expression of HIF-1*α* in the blood vessel after 2 hours of MCAO. Using RECA-1 (endothelial cell marker) immunohistochemical staining method to detect HIF-1*α* expression in endothelial cells of the cortex (Ctx) and striatum (Str) in nonischemic (NI) and ischemic (I) hemispheres after 2 hours of ischemia stroke. (a) Double immunostaining of HIF-1*α* (green) and RECA-1 (red) showed that HIF-1*α* did not colocalize with endothelial cells. *n* = 3 per group. Scale bar = 100 *μ*m. (b) Double immunostaining of HIF-1*α* (green) and RECA-1 (red) showed no expression of HIF-1*α* in bigger blood vessels. Scale bar = 50 *μ*m. *n* = 3 per group.

Using double labeling of HIF-1*α* and the endothelial cell marker RECA-1 [12], we demonstrated that HIF-1*α* was not expressed in endothelial cells (Figure 4(a)) in either striatum (Str) or cortex (Ctx); in addition, HIF-1*α* was not expressed in bigger blood vessels either (Figure 4(b)); this is consistent with our previous study showing that the upregulated HIF-1*α* after acute ischemia stroke was from neurons but not astrocytes or endothelial cells.

## 4. Discussion

The BBB permeability after acute ischemic stroke is critical to determine the outcome of reperfusion by tissue plasminogen activator (tPA) thrombolysis [6] or endovascular mechanical thrombus removal [7]. This critical role of BBB status prompted us to examine BBB injury at the early phase of ischemic stroke in order to provide strategies to improve acute stroke management and show clues for further tPA thrombolysis. This study demonstrated that (1) pretreatment with NADPH oxidase inhibitor apocynin significantly reduced the injury of BBB integrity and disruption of occludin induced by 2 hours of ischemia, (2) pretreatment with apocynin reduced increase of HIF-1*α* expression induced by 2 hours of ischemia, (3) apocynin could also inhibit the MMP-2 upregulation after 2 h ischemic stroke, and (4) HIF-1*α* is not colocalized with bigger blood vessels. The results demonstrate that in the case of acute cerebral ischemia, inhibiting NADPH oxidase can reduce the increase of HIF-1*α* and upregulation of MMP-2, thereby reducing the BBB injury.

NADPH oxidase played a key role in BBB disruption in experimental stroke [16], and its inhibition with inhibitor apocynin improved endothelial function in rat and human blood vessels [18]. Our results showed that apocynin significantly reduced 2 h MCAO-induced BBB injury, providing evidence showing that NADPH oxidase plays a key role in BBB injury not only in the reperfusion stage during which a lot of free radicals were produced but also in the ischemia phase. Since BBB injury was observed in noninfarcted striatum and preoptical area which have moderately reduced cerebral blood flow [20], moderately decreased cerebral blood flow may induce reperfusion-like disruption events such as upregulated reactive oxygen species generations in hypoperfused tissue to accelerate BBB injury [16, 23]. Since our aim is to find a target and strategy to extend the time window and reduce tPA thrombolysis-associated hemorrhage transformation, we investigated the mechanism underlying BBB damage within the thrombolytic time window and we did not investigate the effect of the apocynin on infarction size either.

Apocynin has shown neuroprotective effect after ischemia-reperfusion [17]. For example, coadministration of NADPH plus apocynin provided greater anti-inflammatory and neuroprotective effects on a mouse model of stroke [24], and combination of apocynin with lipoic acid increased the neuroprotection effect on a rat model of stroke [25]. However, apocynin has been shown to improve stroke outcomes with a narrow dose range [26], and apocynin also showed worse outcomes after stroke in aged rats [27]. Therefore, the application of apocynin to reduce ischemia-induced BBB damage and protect the brain must be very careful.

Several factors have shown important roles in regulating HIF-1*α* expression after stroke. For example, HIF-1*α* could be regulated by *β*2-adrenergic receptor after focal ischemia stroke [12]. In addition, C1q contributed to poststroke angiogenesis via the LAIR1-HIF1*α*-VEGF pathway [28], and LncRNA SNHG1 regulated cerebrovascular injury through HIF-1*α*/VEGF after ischemia stroke [29]. In addition, Bu Yang Huan Wu decoction prevented ischemic stroke-induced reperfusion injury in rats by downregulating HIF-1*α* and VEGF and upregulating *β*-ENaC expression [30]. Our results showed that NADPH oxidase inhibition with inhibitor apocynin significantly reduced HIF-1*α* increase induced by 2 hours of MCAO, providing another strategy to regulate HIF-1*α* expression after ischemic stroke.

We have previously found that MMP-2 induction is significantly upregulated in the ventral striatum and preoptic area and the interaction of HIF-1*α* and MMP-2 was critically involved in BBB injury after acute ischemic stroke. Downregulation of HIF-1*α* can reduce BBB injury through inhibiting MMP-2 activity [13]. The results showed that the apocynin significantly reduced the upregulation of MMP-2 induced by 2 h ischemia, suggesting that inhibition of NADPH oxidase-mediated HIF-1*α* upregulation can reduce the BBB injury by reducing the MMP-2 induction.

Our results showed that HIF-1*α* was not colocalized with a bigger vessel, and a previous study has showed that HIF-1*α* was not colocalized with endothelial cells [12], suggesting that HIF-1*α* played an important role in BBB damage, but its upregulation was not induced by the blood vessel after acute ischemia stroke. During the reperfusion stage, the blood vessel may contribute to HIF-1*α* upregulation since HIF-1*α* played a key role in the protection of BBB from reperfusion injury [31, 32]. HIF-1*α* downregulation reduced acute hyperglycemia-induced cerebral hemorrhage transformation in the ischemic hemisphere [33, 34] and decrease BBB injury induced by rat neonatal stroke [35]. Effect of YC-1 treatment on reduction of BBB leakage and reduction of occludin has been shown by our collaborator [12] and us [13].

The findings may provide a new clue to prevent the damage of the integrity of BBB from ischemic damage and to extend the thrombolytic time window of tPA or endovascular treatment and reduce cerebral hemorrhage.

In summary, our results show that during acute cerebral ischemia, inhibiting NADPH oxidase with apocynin reduced BBB damage by regulating HIF-1*α* upregulation and MMP-2 induction. The results gave new clues for preventing ischemic brain injury by protecting the integrity of the BBB and for prolonging the time window of tPA thrombolysis or endovascular treatment and reducing cerebral hemorrhage transformation.

## Data Availability

Data and material will be provided upon request.

## Abbreviations

BBB: Blood-brain barrier
EB: Evan's blue
ECA: External carotid artery
HIF-1*α*: Hypoxia-inducible factor-1 alpha
HT: Hemorrhagic transformation
I: Ischemic hemisphere
ICA: Internal carotid artery
MCAO: Middle cerebral artery occlusion
MMP: Matrix metalloproteinase
NADPH: Nicotinamide adenine dinucleotide phosphate
NI: Nonischemic
POA: Preoptical area
ROI: Region of interest
tPA: Tissue plasminogen activator
TTC: 2,3,5-Triphenyltetrazolium chloride
VEGF: Vascular endothelial growth factor.
